# Stress granule formation helps to mitigate neurodegeneration

**DOI:** 10.1093/nar/gkae655

**Published:** 2024-08-06

**Authors:** M Rebecca Glineburg, Evrim Yildirim, Nicolas Gomez, Genesis Rodriguez, Jaclyn Pak, Xingli Li, Christopher Altheim, Jacob Waksmacki, Gerald M McInerney, Sami J Barmada, Peter K Todd

**Affiliations:** Biological Sciences, Schmid College of Science and Technology, Chapman University, 1 University Drive, Orange, CA 92866, USA; Department of Neurology, University of Michigan, 109 Zina Pitcher Place, BSRB48109-2200, Ann Arbor, MI 4005, USA; Department of Neurology, University of Michigan, 109 Zina Pitcher Place, BSRB48109-2200, Ann Arbor, MI 4005, USA; Department of Neurology, University of Michigan, 109 Zina Pitcher Place, BSRB48109-2200, Ann Arbor, MI 4005, USA; Cell and Molecular Biology Graduate Program, University of Michigan, Ann Arbor, MI, USA; Department of Neurology, University of Michigan, 109 Zina Pitcher Place, BSRB48109-2200, Ann Arbor, MI 4005, USA; Neuroscience Graduate Program, University of Michigan, Ann Arbor, MI, USA; Biological Sciences, Schmid College of Science and Technology, Chapman University, 1 University Drive, Orange, CA 92866, USA; Department of Neurology, University of Michigan, 109 Zina Pitcher Place, BSRB48109-2200, Ann Arbor, MI 4005, USA; Department of Neurology, University of Michigan, 109 Zina Pitcher Place, BSRB48109-2200, Ann Arbor, MI 4005, USA; Department of Neurology, University of Michigan, 109 Zina Pitcher Place, BSRB48109-2200, Ann Arbor, MI 4005, USA; Department of Microbiology, Tumor and Cell Biology, Karolinska Institutet, Stockholm 17165, Sweden; Department of Neurology, University of Michigan, 109 Zina Pitcher Place, BSRB48109-2200, Ann Arbor, MI 4005, USA; Department of Neurology, University of Michigan, 109 Zina Pitcher Place, BSRB48109-2200, Ann Arbor, MI 4005, USA; Veterans Affairs Medical Center, Ann Arbor, MI, USA

## Abstract

Cellular stress pathways that inhibit translation initiation lead to transient formation of cytoplasmic RNA/protein complexes known as stress granules. Many of the proteins found within stress granules and the dynamics of stress granule formation and dissolution are implicated in neurodegenerative disease. Whether stress granule formation is protective or harmful in neurodegenerative conditions is not known. To address this, we took advantage of the alphavirus protein nsP3, which selectively binds dimers of the central stress granule nucleator protein G3BP and markedly reduces stress granule formation without directly impacting the protein translational inhibitory pathways that trigger stress granule formation. In *Drosophila* and rodent neurons, reducing stress granule formation with nsP3 had modest impacts on lifespan even in the setting of serial stress pathway induction. In contrast, reducing stress granule formation in models of ataxia, amyotrophic lateral sclerosis and frontotemporal dementia largely exacerbated disease phenotypes. These data support a model whereby stress granules mitigate, rather than promote, neurodegenerative cascades.

## Graphical Abstract

**Graphical Abstract:**
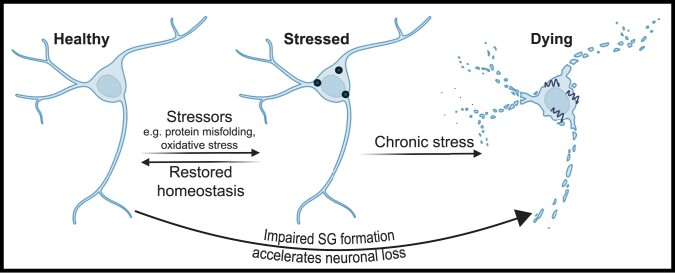

## Introduction

The integrated stress response (ISR) is an adaptive pathway by which any of four different serine/threonine kinases (GCN2, HRI, PERK and PKR) respond to specific types of cellular stress and converge on a canonical translation initiation factor eIF2α. Phosphorylation of eIF2α inhibits ternary complex formation, which reduces global protein translation initiation without impacting elongation and thus creates large unassociated regions of mRNAs that can be recognized by cytoplasmic RNA binding proteins (1,2). This process triggers the formation of stress granules (SGs), which are membraneless organelles that act as processing and sorting sites for mRNA to eventually allow for reinitiation or degradation following stress removal (3). The Ras-GAP SH3 domain binding protein (G3BP) is a key SG nucleator. It binds to both stalled 48S complexes via the 40s ribosome and interacts with single stranded RNAs exposed when translational initiation is blocked (4–6). These mRNAs are subsequently bound by other RNA binding proteins (RBPs) that associate with each other through low complexity domains (LCDs) and indirectly via RNA, creating a ribonucleoprotein complex (3,7,8). Overexpression of G3BP1 alone is enough to promote SG formation in the absence of stress (4,6).

Multiple converging points of evidence suggest that ISR activation and SG formation play prominent roles in neurodegenerative disease, particularly in amyotrophic lateral sclerosis (ALS) and frontotemporal dementia (FTD) (reviewed in (9–11)). One hypothesis is that SG formation accelerates RBP aggregation and that chronic ISR activation or SG protein-associated mutations impede SG disassembly, leading to formation of insoluble inclusions rather than dynamic liquid-liquid phase separation (LLPS) assemblies. In support of this hypothesis, mutations in genes encoding 11 SG proteins are associated with ALS or Alzheimer's disease (11). These mutations tend to be gain-of-function mutations within LCDs that are associated with both translocation of the SG protein from the nucleus to the cytoplasm and protein aggregation (12–15).

A key pathologic aggregate protein in ALS is TDP43, which accumulates in ubiquitin + inclusions in >95% of ALS and approximately half of FTD cases (16). TDP43 is an RBP that primarily resides in the nucleus, where it has key roles in mRNA processing. However, it moves to the cytoplasm in response to cellular stress pathway activation and is a component of some SGs (17–19). Over 40 mutations in the gene encoding TDP43 are linked with ALS and/or FTD. These mutations account for 2–5% of ALS/FTD cases and are overwhelmingly located within the C-terminal LCD. TDP43 mutants mis-localize to the cytoplasm, disrupt SG disassembly, and form persistent cytoplasmic protein aggregates (12,19,20).

G_4_C_2_ hexanucleotide repeat expansions within the *C9orf72* gene are the most common mutations responsible for inherited ALS and FTD, accounting for up to 40% of familial disease (21,22). These expansions produce arginine-rich dipeptide repeats (DPRs) through a process known as repeat associated non-AUG (RAN) translation (23–27). DPRs accelerate neurodegenerative pathology through a variety of mechanisms, one of which is associating with SGs and forming insoluble aggregates. Moreover, ISR activation enhances RAN translation and DPR production, and G_4_C_2_ repeat expression elicits ISR activation (23–24,28–31).

SG induction itself can also directly elicit neurodegeneration. ISR activation in the absence of a disease background recapitulates key ALS phenotypes including accumulation of TDP43 aggregates (17,32,33). Repeated formation of SGs in the absence of ISR activation, achieved by using optogenetics to artificially dimerize SG nucleator G3BP, also triggers formation of TDP43 aggregates and enhanced death in neurons (33). These studies suggest that while mutant RBPs likely accelerate the rate of neurodegeneration, frequent ISR activation alone is sufficient to cause it.

If SG formation contributes to neurodegenerative disease, then inhibiting SG formation could be a viable strategy to prevent neurodegeneration. Indeed, a number of studies have used inhibitors of the ISR to suppress neurodegenerative phenotypes in mice and *Drosophila* (17,34–37). However, blocking the ISR also prevents eIF2α triggered protein translational inhibition and activation of a stress response cascade that independently elicits cell death (3,17,38,39), making it difficult to tease out the contributions of SGs specifically to neurodegeneration.

Viral proteins offer an intriguing alternative approach. Viruses have evolved methods to escape the ISR because its activation would otherwise prevent viral protein synthesis. Semliki Forest virus (SFV), escapes the ISR through generation of a nonstructural protein 3 (nsP3) which binds G3BP and prevents SG formation (40–45). Cells infected with WT SFV, but not with a mutant nsP3 SFV, were unable to form SGs in response to sodium arsenite (NaArs) and pateamine A, indicating nsP3 can inhibit both eIF2α dependent and independent SG formation (39,44). nsP3 binds NTF2-like domains of G3BP1 through its two FGDF domains, such that each FGDF domain can sequester one G3BP1 dimer to form a poly-complex with four G3BP molecules (40). Binding to G3BP creates a network of nsP3:G3BP complexes that assist in viral replication, and promotes viral protein synthesis by recruiting 40S ribosomes to viral RNA (40,45,46). Interestingly, a 31 amino acid fragment containing both FGDF domains (nsP3-31) is sufficient for binding G3BP1, and these domains are likewise necessary to inhibit SG formation by nsP3 (42–44).

Here, we took advantage of this nsP3-31 fragment to inhibit SG formation in ALS/FTD models and assess whether preventing SGs alleviated toxicity. While nsP3-31 effectively inhibited SG formation induced by a variety of mechanisms, it was detrimental in several neurodegenerative disease models, suggesting that SG formation may be beneficial at least initially in these contexts. These findings have important implications for future therapeutic development targeting these pathways in neurodegenerative disorders.

## Materials and methods

### Plasmids, primers and flies

pEGFP-nsP3-C-term-WT and pEGFP-nsP3-C-term-F3A were previously generated (40,42,44). pUASTattB and pUASz1.1 plasmids for making transgenic flies were generated using restriction enzyme cloning. pUASTattB plasmids were generated by inserting mApple and downstream C-term sequences between NotI and KpnI in pUASTattB parental vector (47) (Supplementary Table S1). pUASz1.1 plasmids were generated by inserting mApple and downstream C-term sequences between NotI and XbaI in pUASz1.1 (DGRC Stock 1433; https://dgrc.bio.indiana.edu//stock/1433; RRID:DGRC_1433). Primers were synthesized from IDT. Specifics about flies used in this study can be found in Supplementary Table S2.

### Cell studies

Transfections were performed as previously described (23). In brief, HEK293Ts were seeded at 1 × 10^5^ cells/ml and transfected with 3:1 FuGene HD 24 h later. For Nluc assays: a 1:1:10 ratio of Nluc reporter:FFluc reporter: nsP3 effector plasmid was used. For ICC, a 1:1 ratio of WT or mut nsP3 plasmid was co-transfected with either Poly(I:C) or Nluc reporter. Twenty-four hours post transfection, cells lysates were harvested for western blots, SUnSET assay, and Nanoluciferase assays as previously described or fixed in 4% PFA for immunocytochemistry (23). The following antibodies were used for ICC: ms anti-DDX3 (1:250) (SCBT sc-81247), ms anti-G3BP1 (1:250) (BD Bioscience BDB611127), Goat anti-mouse Alexa Fluor 633 (1:500) (Invitrogen A-21146). The following antibodies were used at 1:1000 for HEK293T lysate western blots: ms anti-B-tubulin (DSHB E7), and ms anti-puromycin (Millipore MABE343), rb anti-GFP (Sigma 11 814 460 001) and rb anti-PERK (CST 3192S). Blots were detected using HRP conjugated goat anti-mouse and goat anti-rabbit secondaries.

### Rough eye scoring and eye width measurements

Male flies containing the neurodegeneration causing transgene were crossed to GMR-Gal4 females at either 29C (UAS-(CGG)_90_-EGFP, EGFP-TDP43, TDP43-M337V), 25C ((GGGGCC)_28_-EGFP) or 18C (GR_100_-EGFP). One eye from each progeny was assayed for a rough eye phenotype or eye width measurement. Rough eye phenotype assays were performed according to previously published methods (48). Fly eye images were taken using a Leica M125 stereomicroscope and a Leica K3C equipped with LAS X manual z-stacking software. Eye widths were measured using LAS X software.

### Survival assay

Fly longevity assays were performed according to previously published methods (Singh *et al.*, 2021). Briefly, flies were separated by sex at 0–48 h post eclosion, and put on food containing RU-486. Every 48 h, dead flies were counted and surviving flies were moved to fresh food containing RU-486. Serial heat shock survival assays were performed similarly with the addition of exposing flies to 36°C heat shock for 2 h every 48 h.

### Larval qRT-PCR and western blots

Ten third instar wandering larvae for each genotype were collected and stored at −80°C prior to lysis. Larvae were resuspended and homogenized in trizol according to manufacturer's protocol (Invitrogen Trizol) with the following modifications. RNA was precipitated in isopropanol overnight at −20°C. RNA was resuspended in 50 ul H_2_O and column purified prior to cDNA synthesis.

500 ng of RNA/sample was used to make cDNA using iScript cDNA synthesis kit according to manufacturer's protocol (Bio-Rad). cDNA abundance was measured using 300 nM of indicated primers and a iQ5 qPCR system (Bio-Rad).

For fly westerns, five third instar wandering larvae/genotype were lysed in RIPA + miniComplete protease inhibitor cocktail, hand homogenized on ice 20×, and centrifuged at 12 000 × g and 4C for 5 minutes. Lysates were transferred to new tubes, homogenized through a 28 G insulin syringe 8×, and boiled in 1× LB for 5 min. Samples were run on 12% Bis–Tris gels, and transferred according to previously published protocols (Singh *et al.*, 2021). Membranes were blotted with rb anti-mCherry (ThermoFisher PA5-34974) 1:1000 (for mApple), ms anti-B-tubulin (DSHB E7) 1:1000, and ms anti-Flag M2 (Sigma F1804), 1:1000, and detected using HRP conjugated secondaries Gt-anti-ms, and Gt-anti-rb 1:5000.

### Larval brain dissections, drug treatments and fluorescence imaging

Brains from third instar wandering larvae were dissected in 1× PBS, and transferred to Shields and Sang M3 insect medium, with 2% FBS and 2.5% fly extract (Gareau, C., Plos One, 2013). Dissected brains were incubated at 42°C for 1 h, or at 25°C with either 500 μM NaArs (2 h), 10 uM Thapsigargin (1.5 h) or untreated (2 h). Brains were then washed in 1× PBS, fixed for 20 min in 4% PFA at RT, washed 3× for 5 min in 1× PBS, then incubated overnight at 4°C in 1× PBS + 0.1% Tween with 1 ug/ml DAPI, and mounted in Prolong Gold. For rin-sfGFP samples, Z-stacks containing 1 uM sections were imaged on an Olympus FV100 using 40× oil objective, 2× zoom and processed using ImageJ. 1 slice/brain is depicted in representative images. TDP43 samples were imaged on an Zeiss LSM 880 Confocal Microscope using 63× oil objective at 1.3 zoom, 1 um pinhole, and processed using ImageJ as above. Foci over a specified threshold were counted as either small (5–15 pixels), medium (15–25 pixels) or large (25–35 pixels).

### Eclosion assay

15 Actin/Cyo females and 5 mApple-x/mApple-x males were put on apple agar plates with wet yeast for 24 h. Embryos were collected at 24 h, counted under a microscope, and put on fresh 10% SY food. Number and genotype of eclosed flies were recorded 10–15 days later. The proportion of mApple expressing/total number eclosed was used to determine eclosion rates with each genotype, with an a priori expected ratio of 1:1 mApple expressing: Cyo, mApple not expressing.

### Climbing assays

Male flies were separated 48 h post eclosion and transferred to fresh food every 2 days. At indicated ages, individual flies were put into narrow vertical channels ∼9 cm high in custom made monitors. The monitors were dropped to collect flies at the bottom of the channels, and the time required for the flies to reach 75% of the channel height is determined with a camera. Each experiment is repeated three times and the average time for each fly is taken.

### Primary neuron transfection

Primary mixed cortical neurons were dissected from embryonic day 19–20 Long-Evans rat pups and cultured at 0.6 × 10^6^ cells/ml in 96-well cell culture plates (TPP) coated with laminin (Corning) and d-polylysine (Millipore) (48–51). At *in vitro* day (DIV) 3–4, neurons were transfected with 0.2 μg DNA and 0.5 μl Lipofectamine 2000 (ThermoFisher) per well, per the manufacturer's protocol, with the exception that cells were incubated with Lipofectamine/DNA complexes for 20 min at 37**°**C before rinsing. Automated longitudinal fluorescence microscopy began 24 h post-transfection for 10 days, as previously described (49–53). Briefly, images were acquired by an inverted Nikon Ti microscope equipped with a 20× objective lens, a PerfectFocus system, a Lambda 421 multichannel LED with 5 mm liquid light guide (Sutter), and either an Andor iXon3 897 EMCCD camera or Andor Zyla4.2 (+) sCMOS camera. All stage, shutter and filter wheel movements were carried out by custom code written in publicly available software (μManager, ImageJ).

### Primary neuron imaging and puncta determination

Primary cortical neurons were isolated and plated onto glass coverslips, then transfected on DIV as described above. Forty-eight hours post-transfection, neurons were exposed to 250 mM sodium arsenite or DMSO in PBS for 30 min at 37°C, then washed twice with PBS before fixing with 4% paraformaldehyde in PBS for 10 min at RT, and permeabilizing with 0.1% Triton-X-100 in PBS for 20 min at RT. The cells were neutralized with 10 mM glycine in PBS at RT before blocking in PBS containing 0.1% Triton, 2% FCS and 3% BSA, for 1 h at RT. Detection was accomplished by incubating with anti-ATXN2 primary antibodies (diluted 1:500 in blocking solution, BD Biosciences 611 378) overnight at 4°C. Samples were rinsed 3 times in PBS, incubated with Alexa 647 labeled donkey anti-rabbit secondary antibodies (1:250 in blocking solution; Invitrogen A32795TR) for 1 h at RT, then rinsed 3 more times in PBS containing a 1:1000 dilution of Hoechst 33 258 nuclear dye (Invitrogen H3569) prior to confocal fluorescence imaging using a Nikon NSPARC confocal microscope. Sum intensity projections were created for each image, and regions of interest corresponding to individual neurons were drawn for GFP-positive soma. Within each region of interest, the coefficient of variability (CV) was calculated as the ratio of standard deviation of signal intensity to the mean signal intensity for the ATXN2 channel.

## Results

**Figure 1.**
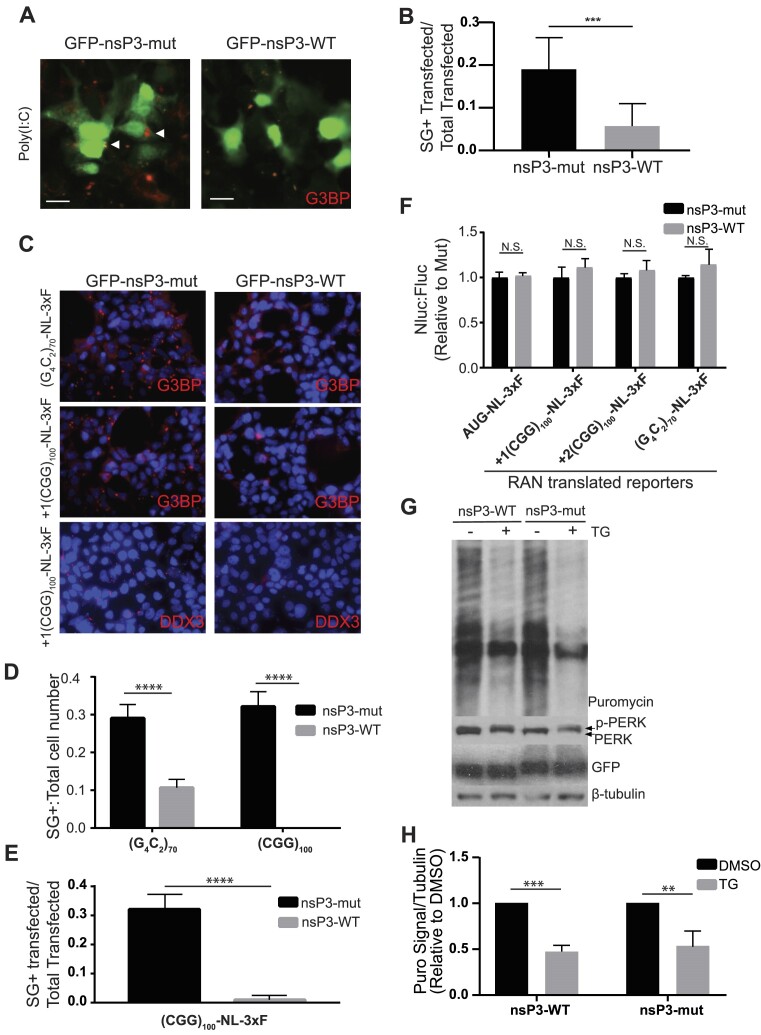
nsP3 inhibits SG formation caused by GC-rich repeats, but has no effect on translation. (**A**) HEK 293T cells co-transfected with WT or mut nsP3 and poly(I:C). (**B**) Quantification of G3BP SG + cells in (A). Bars represent proportion ± 95% C.I. mut nsP3 *n* = 117, WT nsP3 *n* = 124. (**C**) HEK 293T cells co-transfected with WT or mut nsP3 and the indicated Nluc-3xF reporters. G3BP and DDX3 are used as SG markers. (**D**) Quantification of G3BP SG + cells in (**C**). Bars represent proportion ± 95% C.I.s (G4C2)70 + mut nsP3 *n* = 622, (G4C2)70 + WT nsP3 *n* = 731, (CGG)100 + mut nsP3 *n* = 538, (CGG)100 + WT nsP3 *n* = 685. (**E**) Quantification of DDX3 SG + cells in (C). Bars represent proportion ± 95% C.I. (CGG)100 + mut nsP3 *n* = 324, (CGG)100 + WT nsP3 *n* = 314. (B, D, E) Two tailed fisher's exact test with Bonferonni correction for multiple comparisons (D), ***P< 0.001, ****P< 0.0001. (**F**) Nanoluciferase expression relative to FireFly luciferase (FFLuc) of indicated Nanoluciferase (Nluc)-3xF reporters co transfected with EGFP-nsp3 WT or EGFP-nsP3 mut. Bars represent mean ± SEM. Two-way ANOVA and Holm-Sidak unpaired *t*-tests, *n* = 6. (**G**) Western blot and SUnSET Assay (puromycin) of HEK 293Ts transfected with EGFP-WT nsP3 or EGFP-mutant nsP3 and treated with either DMSO (control) or Thapsigargin (TG). B-tubulin = loading control, p-PERK = control for TG ISR induction. (**H**) Quantification of SUnSET assay in (G). Bars represent mean ± standard deviation, Welch's *t*-tests, *n* = 3, *P< 0.05, **P< 0.01.

A GFP-nsP3-31 fusion protein was previously shown to bind tightly to G3BP (44). To determine whether this fusion protein inhibits canonical SG formation, we co-transfected either a WT EGFP-nsP3-31 (nsP3-WT) or a mutant EGFP-nsP3-31 (nsP3-mut) in which the two FGDF domains contain single phenylalanine (F) to alanine (A) mutations that prevent binding to G3BP (42,44). To assess whether nsP3-WT could inhibit ISR-induced SGs, we applied the dsRNA mimic poly(I:C), which activates the ISR through the dsRNA protein kinase PKR. Poly(I:C) robustly induced SGs when transfected into HEK293T cells (Supplementary Figure S1A). NsP3-WT, but not nsP3-mut, inhibited poly(I:C) induced SGs (Figure 1A, B).

Next, we assessed whether nsP3-WT could inhibit SGs induced by a neurodegenerative trigger. We previously showed that Fragile-X-associated tremor ataxia syndrome (FXTAS) CGG-repeat and ALS/FTD associated G_4_C_2_-repeat containing RAN reporters activate the ISR and elicit eIF2α-dependent SGs (23). Co-transfecting nsP3-WT with either FXTAS associated (CGG)_100_ repeats or ALS/FTD associated (G_4_C_2_)_70_ repeats significantly inhibited RAN reporter-induced SGs compared to nsP3-mut (Figure 1C–E). Despite inhibiting SG formation, the presence of nsP3-WT had no effect on canonical translation of AUG-nanoluciferase or firefly luciferase reporters or RAN translation of both CGG and G_4_C_2_ repeat containing reporters (Figure 1F and Supplementary Figure S1B, C). When cells were stressed with the ER calcium pump inhibitor, thapsigargin (TG), which activates PERK, neither nsP3-WT or nsP3-mut affected PERK activation or global translational inhibition (Figure 1G, H). Together, these data suggest that nsP3 can be used to selectively inhibit SG formation without altering ISR-induced translational suppression.

**Figure 2.**
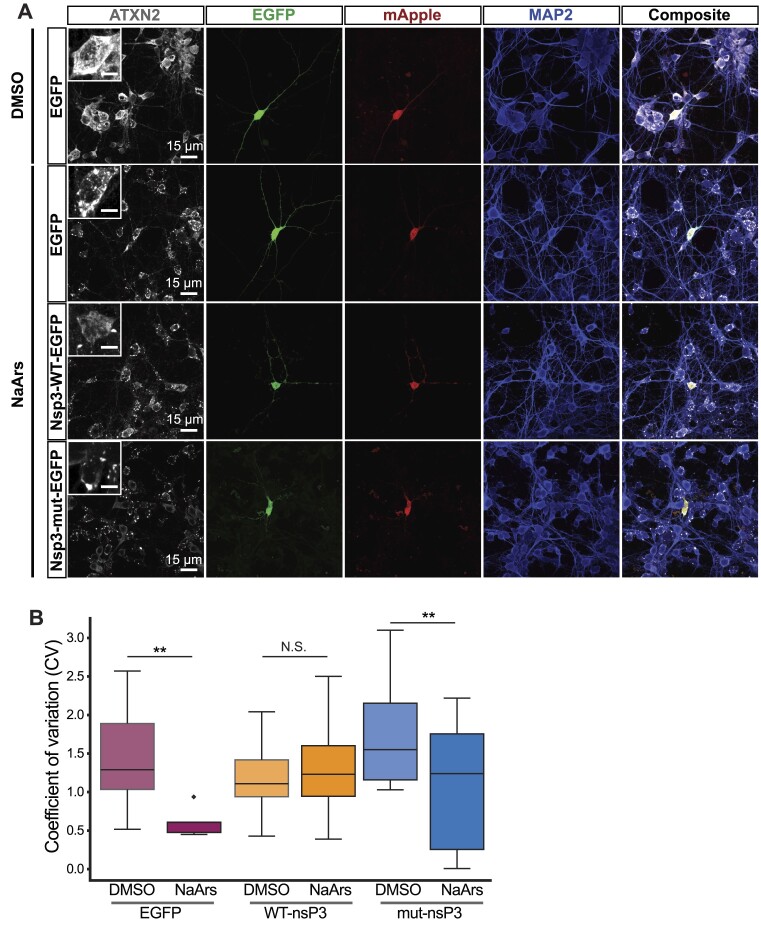
nsP3 reduces SG formation in neurons. (**A**) Primary rat cortical neurons treated with 250μM NaArs for 30min prior to immunostaining for the SG marker ATXN2. (**B**) Quantification of coefficient of variation (CV) of granular ATXN2 staining in (A). *P= 0.021, **P= 0.010, ns: *P*> 0.05; Welch's unpaired *t*-test. (A) *N* = 14–19 cells/condition, pooled from three replicates.

To better understand the role of SGs in mammalian neurodegeneration, we investigated whether nsP3 could influence SG formation in rat cortical neurons. Consistent with prior studies, we confirmed the formation of ATXN2 and G3BP1 positive SGs in rodent primary cortical neurons in response to NaArs (54,55) (Figure 2A and Supplementary Figure S2). In comparison to inactive nsP3-mut, nsP3-WT significantly reduced the appearance of SGs in transfected neurons, as judged by a single-cell measure of ATXN2 granularity (55,56) (Figure 2B).

**Figure 3.**
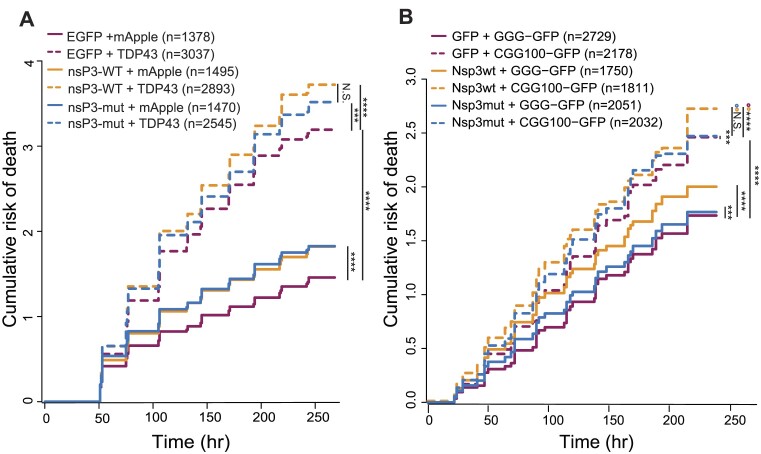
nsP3 does not enhance survival in primary neurons expressing TDP43 or trinucleotide repeats. (A, B) Automated microscopy and survival analysis of neurons expressing (**A**) TDP43-mApple and nsP3-WT, nsP3-mut, or mApple EGFP control or (**B**) (CGG)100-GFP and nsP3-WT, nsP3-mut or mApple EGFP control. Data inA pooled from three biological replicates, each with eight technical replicates/condition. Data in (B) are combined from four biological replicates, each with eight technical replicates/condition. Cox proportional hazards analysis, ***P< 0.001, ****P< 0.0001.

We next determined whether nsP3-WT expression could enhance survival in two established disease models: a TDP43 overexpression ALS/FTD model (TDP43-mApple), and a neuronal model of FXTAS ((CGG)_100_-EGFP) (57,58). As expected, overexpressing TDP43-mApple significantly increased the risk of death in comparison to mApple alone (Figure 3A). Co-expression of nsP3-WT failed to rescue toxicity arising from TDP43-mApple, however, and instead increased the risk of death further. Similar results were observed with nsP3-mut overexpression. In addition, neither nsP3-WT nor nsP3-mut prevented neurodegeneration in our FXTAS rat primary neuron model (Figure 3B). Here, overexpression of (CGG)_100_-EGFP resulted in toxicity that was unaffected, and even somewhat exacerbated, by co-expression of nsP3-WT or nsP3-mut co-expression. Further, expression of nsP3-WT and nsP3-mut displayed subtle increases in the risk of death compared to EGFP control, suggesting intrinsic toxicity associated with the C-terminal 31 residues of nsP3 regardless of its ability to bind G3BP (Figure 3A, B). These data suggest nsP3-31-WT, despite reducing SG formation in neurons (Figure 2) fails to mitigate neurodegeneration associated with TDP43 or CGG repeat expression.

**Figure 4.**
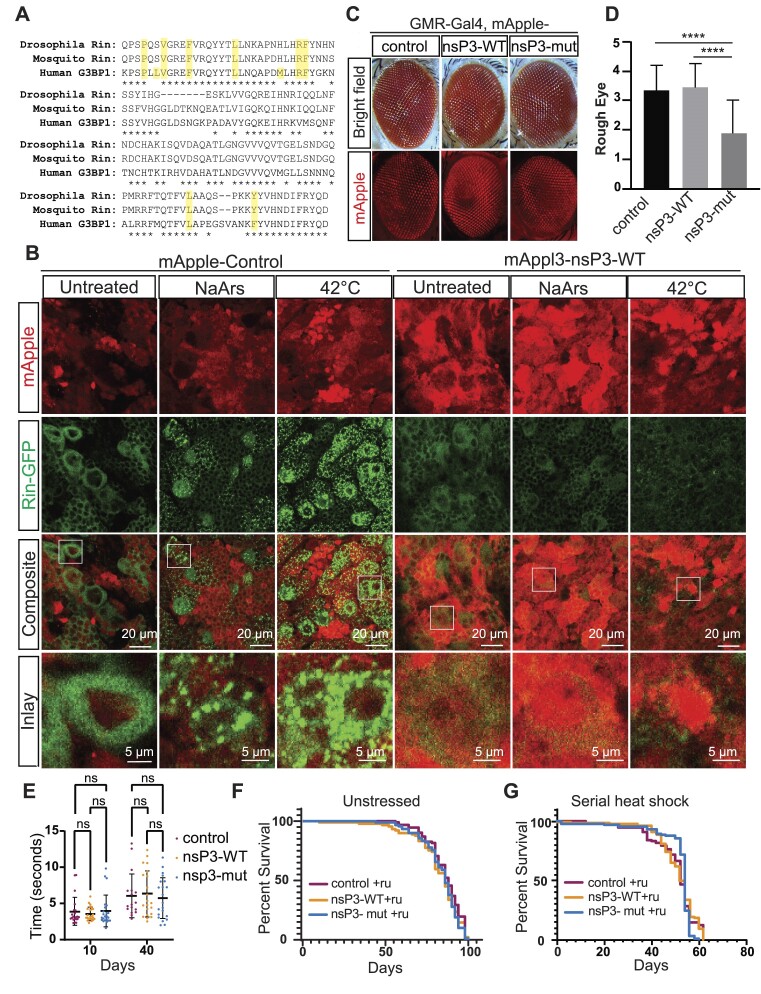
Impact of nsP3 expression in Drosophila. (**A**) Schematic of Drosophila rin, *Aedes albopictus* rin and human G3BP1 NTF2-like domains. Asterisks indicate homology, yellow highlights denote nsP3 interacting regions. (**B**) Representative images of third instar larval brains expressing rin-sfGFP, and Actin-Gal4, UAS-mApple-control (left) or Actin-Gal4, UAS mApple-nsP3 WT (right) untreated, 2 h in 500uM NaArs, or 1 h at 42C. (**C**) Representative images of eyes from flies coexpressing GMR-Gal4 with mApple-control,mApple-nsP3-WT, or mApple-nsP3-mut. (Top) Bright field, (bottom) mApple expression. (**D**) Quantification of rough eye phenotypes of males from C (control *n* = 23, nsP3-WT *n* = 33, nsP3-mut *n* = 26). One-way ANOVA and Tukey's multiple comparisons test. ****P< 0.0001. (**E**) Climbing time showing mean time ± stdev to climb 6.5 cm for male D42-Gal4, mApple control, nsP3-WT or nsP3-mut flies, conducted at indicated days post-ecolosion at 25C. *n* ≥ 17. Two-way ANOVA and Tukey's multiple comparison test. (**F**) Survival assay of Tub5-GS, mApple control, nsP3-WT, or nsP3-mut flies exposed to RU-486 at 24C. (control *n* = 92, nsP3-WT *n* = 90, nsP3-mut *n* = 89). Logrank Mantel–Cox test. (**G**) Survival assay Tub5-GS, mApple control, nsP3-WT or nsP3-mut flies exposed to RU-486 and serially heat shocked (incubated at 24°C then heat shocked for 2 h at 36°C every 48 h) (control *n* = 90, nsP3-WT *n* = 89, nsP3-mut *n* = 88). (F, G) Log-rank Mantel–Cox test, no significance observed between genotypes.

To determine if nsP3 has any effects in an *in vivo* setting, we turned to a *Drosophila* model system. *Drosophila* homologue Rin NTF2-like domains are only 60.6% identical to human G3BP1 and share identical or similar homology for 8 out of the 10 known nsP3 interacting sites (Figure 4A) (40); however nsP3 has also been shown to bind *Aedes albopictus* (mosquito) Rin (59,60), and *Drosophila* Rin NTF2 domains are 93% identical to mosquito Rin, including identical amino acid identity at all 10 nsP3 interacting sites (Figure 4A). Thus, we reasoned that nsP3 could bind Rin and also block SGs in *Drosophila*. We generated UAS-mApple-nsP3-WT (nsP3-WT) and UAS-mApple-nsP3-mut (nsP3-mut) *Drosophila* lines and compared overall expression levels to an UAS-mApple line (control). nsP3-WT and nsP3-mut lines expression was comparable in terms of mRNA and protein levels, but both were expressed at lower levels than mApple alone despite use of a shared psi31 insertion locus (Supplementary Figure S3A–C). As expression levels were variable within and between a given genotype, we used both controls in our experiments. Importantly, when we coexpressed nsP3-WT with Rin-GFP, we observed no effect on total Rin-GFP protein levels compared to coexpression with nsP3-mut or mApple control, suggesting any differences observed between these three fly lines is not due to expression levels of Rin protein (Supplementary Figure S3D).

To determine if nsP3-WT interacts with Rin and inhibits SG formation in flies, we dissected brains from control, nsP3-WT and nsP3-mut third instar larvae expressing an endogenously GFP-tagged *rin* (61) and exposed them to specific stress conditions. Both Actin-Gal4 expressing control and nsP3-mut lines showed strong SG formation in the central brain after heat shock or treatment with NaArs or thapsigargin (Figure 4B and Supplementary Figure S4A, B). In contrast, Actin-Gal4/nsP3-WT brains had no detectable SGs under any of these conditions. SGs were visible in cells in the optic lobes, but were rarely seen in the ventral nerve cord for all genotypes under all conditions (data not shown).

Expressing a variety of factors associated with neurodegeneration in the fly eye can elicit a rough-eye phenotype associated with ommatidial degeneration. GMR-Gal4 expression of nsP3-WT in isolation did not induce eye degeneration compared to control (Figure 4C, D). While both the control and nsP3-WT expressing fly eyes scored significantly higher than nsP3-mut in males, no differences were observed between nsP3-WT and control females (Figure 4C, D and Supplementary Figure S4C). Further, using a GFP-based apoptotic reporter (62), we did not observe any apoptosis in fly eyes expressing control, nsP3-WT, or nsP3-mut compared to an apoptosis positive control fly expressing GMR-reaper (63) (Supplementary Figure S4D).

ALS is a motor neuron disease, and thus we sought to determine if inhibiting SG formation specifically in motor neurons impacted motor neuron function. We used the motor neuron driver D42-Gal4 (64) to express nsP3-WT, nsP3-mut or mApple control and performed climbing assays on young and old flies. We observed a significant increase in climbing time across all genotypes with age, but did not observe any significance differences in climbing deficits between genotypes of the same age (Figure 4E and Supplementary Figure S4E).

To assess the impact of SG formation inhibition on longevity, we utilized the Tub5-Geneswitch system to activate nsP3-WT, nsP3-mut, or mApple control expression ubiquitously post eclosion. Under non-stressed conditions at 24C, nsP3-WT alone had no impact on longevity in adult flies in comparison to nsP3-mut or control flies (Figure 4F). Similarly, Tub5-GS/nsP3-WT lines which were not treated with the gene-activating agent RU-486 lived similar lifespans compared to lines where Ru-486 activated nsP3-WT expression (Supplementary Figure S4F compared to Figure 4F). nsP3-WT expression also had no effect on eclosion proportions when expression was driven ubiquitously during development using an Actin-Gal4 driver (Supplementary Figure S4G). Taken together, these data suggest nsP3 expression under non-stress conditions is not harmful.

To assess whether nsP3 might affect survival under conditions of serial stress exposure, we exposed flies expressing nsP3-WT, nsP3-mut, or mApple in neurons to serial heat shock (2 h at 36°C every 2 days). This led to a significant decrement in survival in all fly lines; however, this effect was independent of genotype (Figure 4G compared to 4F). Thus, inhibition of SG formation even in the setting of serial stress response activation has no impact on survival.

We next investigated whether drivers of neurodegeneration associated with altered SG dynamics might respond to nsP3 expression. TDP43 aggregation in particular is a common feature in both ALS and FTD, and TDP43 itself is a SG protein. Moreover, pathogenic TDP43 cytoplasmic aggregation in ALS may be driven in part by SG formation (17,32,33,65,66). To investigate this in our model system, we co-expressed mApple control, nsP3-WT, or nsp3-mut with EGFP-TDP43 in third instar larval brains using the neuroblast driver Asense-Gal4. This driver was chosen as previous attempts co-expressing nsP3-WT with EGFP-TDP43 using the Actin-Gal4 driver was lethal prior to the third instar larval stage, and observations in Rin-GFP flies indicated SG forming potential is abundant in neuroblasts (Figure 4A). Under non-stressed conditions, EGFP-TDP43 expression was largely diffuse with occasional aggregates (Supplementary Figure S5A). nsP3-WT did not alter EGFP-TDP43 expression levels or aggregates compared to nsP3-mut or mApple control expressing lines under non-stressed conditions. Upon NaArs treatment, the diffuse EGFP-TDP43 signal decreased and brighter puncta were observed (Supplementary Figure S5B). nsP3-WT had no effect on number or size of EGFP-TDP43 foci in brains compared to either the nsP3-mut or mApple control (Supplementary Figure S5C–D) This suggests nsP3-WT is unable to prevent stress-induced TDP43 aggregation.

We next asked whether nsP3-WT could alleviate neurodegenerative phenotypes in four *Drosophila* ALS models: two TDP43 models (overexpression of EGFP-TDP43 or the ALS-associated TDP43 (M337V) mutant, which enhances TDP43 cytoplasmic mislocalization, disrupts SG dynamics and causes more rapid neurodegeneration in *Drosophila* (20,67–69)), and two *C9orf72* RAN translation models ((GGGGCC)_28_-GFP, and (GR)_100_), both of which express glycine–arginine DPRs that form large GR aggregates that cause extensive neurodegeneration and impair SG dynamics (20,24,70,71)).

**Figure 5.**
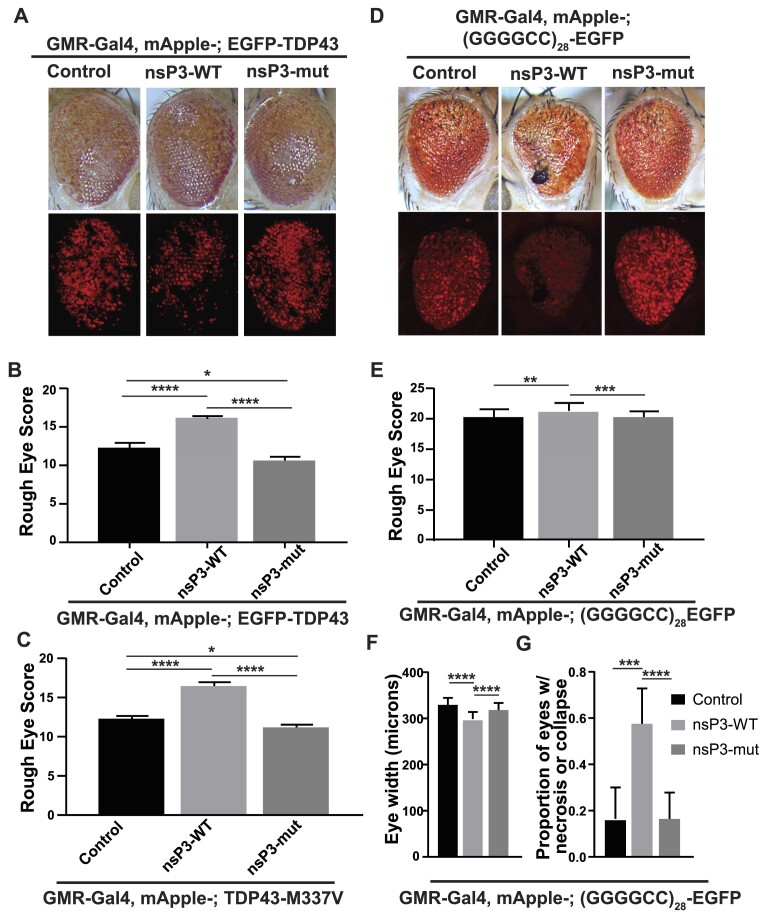
nsP3 enhances toxicity in ALS fly models. (**A**) Representative images of male fly eyes expressing EGFP-TDP43 under GMR-GAL4 expression with control, nsP3-WT or nsP3- mut. (**B**) Quantification of rough eye phenotypes of males from A (control *n* = 34, nsP3-WT *n* = 68, nsP3-mut *n* = 74). (**C**) Quantification of rough eye phenotypes of males expressing TDP43-M337V under GMR-GAL4 expression with control, nsP3-WT or nsP3-mut (control *n* = 33, nsP3-WT *n* = 33, nsP3-mut *n* = 52), ****P< 0.0001. (**D**) Representative images of male fly eyes expressing (GGGGCC)28-EGFP under GMR-GAL4 expression with control, nsP3-WT, or nsP3-mut. (**E**) Quantification of rough eye phenotypes of males from D (control *n* = 44, nsP3-WT *n* = 33, nsP3-Mut = 61). (**F**, **G**) Quantification of (D, F) eye width (control *n* = 23, nsP3-WT *n* = 23, nsP3-mut *n* = 21), and (G) proportion of necrosis positive eyes (control *n* = 44, nsP3-WT *n* = 33, nsP3-Mut = 61). (B, C, E) One-way ANOVA and Tukey's multiple comparisons test. (F) Two way ANOVA (see Supplementary Figure S5D) with Tukey's multiple comparisons test. *P< 0.05, **P,0.01, ***P< 0.001, ****P< 0.0001. (G) Fischer's exact test with Bonferonni correction for multiple comparisons. *P< 0.01667, **P< 0.01, ***P< 0.001, ****P< 0.0001.

In GMR-Gal4 driven models that express EGFP-TDP43 in the fly eye, nsP3-WT enhanced the rough eye phenotype compared to both control and nsP3-mut (Figure 5A, B and Supplementary Figure S6A). Similar effects were observed for both (GGGGCC)_28_-GFP and TDP43(M337V) expressing lines (Figure 5C–E and Supplementary Figure S6B, C). nsP3-WT also reduced eye width and enhanced necrosis in (GGGGCC)_28_-GFP lines compared to control and nsP3-mut (Figure 5F, G and Supplementary Figure S6D, E) flies. However, nsP3-WT had no effect on the (GR)_100_ expressing line or a FXTAS RAN translation model (Supplementary Figure S6F, G). Together these results suggest that inhibiting SG formation and TDP43 aggregation is not beneficial, even in disease models where SG dynamics are known to be disrupted or where SG formation itself contributes to aberrant protein aggregation.

**Figure 6.**
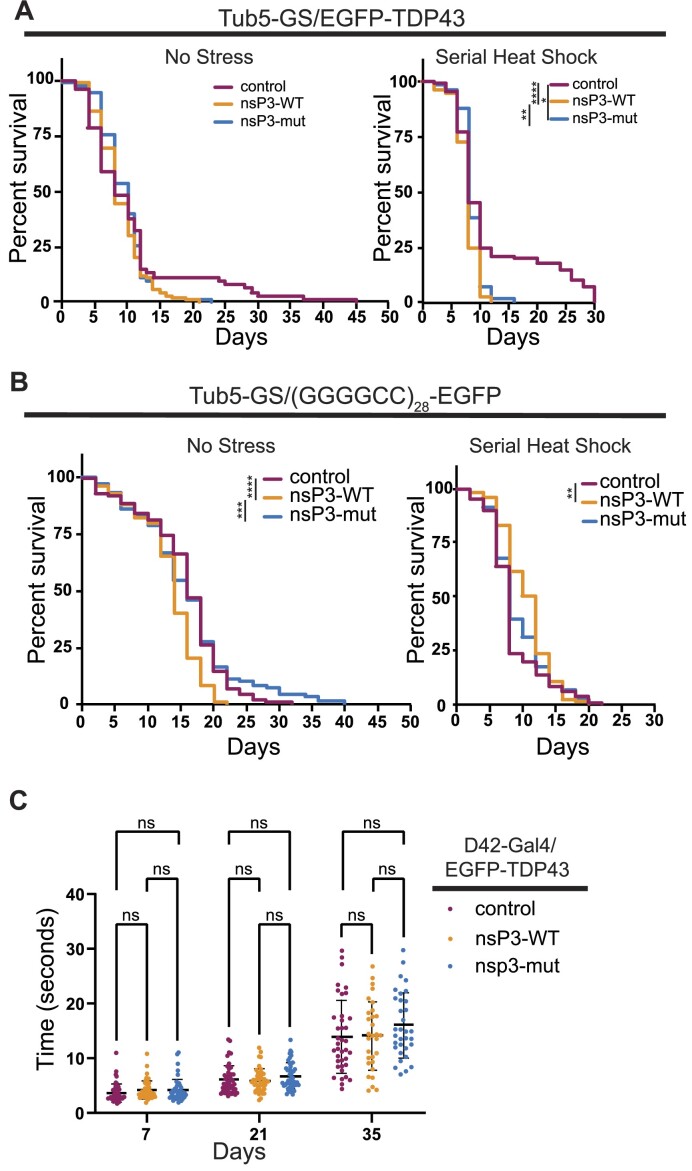
nsP3 does not enhance survival in ALS fly models. (**A**) Survival curves of Tub5-GS/EGFPTDP43 coexpressed with indicated genotypes either at (left) constant 29°C (control *n* = 75, nsP3-WT *n* = 111, nsP3-mut *n* = 112) or (right) serial heat shock (2 h every 48 h at 36°C) (control *n* = 81, nsP3- WT *n* = 85, nsP3-mut *n* = 76). (**B**) Survival curves of Tub5-GS/(GGGGCC)28-EGFP coexpressed with indicated genotypes either at (left) constant 29°C (control *n* = 87, nsP3-WT *n* = 84, ns3P3-mut *n* = 109), or (right) serial heat shock (2 h every 48 h at 36°C) (control *n* = 111, nsP3-WT *n* = 106, nsP3-mut *n* = 106). Log-rank Mantel–Cox test with Bonferroni corrections for multiple comparisons. *P< 0.0125, **P< 0.01, ***P< 0.001, ****P< 0.0001. (**C**) Climbing assay showing mean time ± stdev to climb 6.5 cm for D42-Gal4, UAS-EGFP-TDP43 male flies coexpressing mApple control, nsP3-WT or nsP3-mut conducted at indicated days post-ecolosion at 25°C. *n* ≥ 30, Two-way ANOVA and Tukey's multiple comparison test.

We next investigated whether nsP3-WT could enhance longevity in stress and nonstress conditions in two ALS *Drosophila* models: a TDP43 overexpression fly, and *C9orf72* RAN translation model (GGGGCC)_28_-GFP) (67,68,70). Both lines have significantly reduced survival relative to control lines (Supplementary Figure S7A) (70). nsP3-WT had no effect on longevity in flies expressing EGFP-TDP43 compared to flies expressing nsP3-mut or mApple control under nonstress conditions (Figure 6A, *left*). Similarly, in this same TDP43 overexpression background, expressing nsP3-WT in the presence of serial heat shock exposure was detrimental compared to nsP3-mut and mApple control (Figure 6A, *right*). In the (GGGGCC)_28_-GFP background, nsP3-WT significantly reduced survival under non-stressed conditions (Figure 6B, *left*). Conversely, under serial heat shock exposure, nsP3-WT significantly improved survival of (GGGGCC)_28_-GFP flies relative to control, but not relative to nsP3-mut (Figure 6B, *right*). Whether this discrepancy is biologically relevant is unclear.

FXTAS-associated CGG repeats have also been implicated in ISR activation, and expression of CGG repeats in flies produces polyglycine aggregates (23,72). As with other models, nsP3-WT had no effect on survival of the FXTAS model fly expressing (CGG)_90_-EGFP (Supplementary Figure S7B).

To determine if nsP3-WT impacts motor neurons specifically, we coexpressed EGFP-TDP43 with either control, nsP3-WT, or nsP3-mut using the motor-neuron specific driver D42-Gal4 (64). Motor neuron specific TDP43 expression leads to a more rapid functional decline in climbing compared to control flies (73). While we observed an age-dependent decrease in climbing ability within genotypes, we observed no differences in climbing ability across genotypes at all timepoints (Figure 6C and Supplementary Figure S7C). Together, these findings suggest toxicity in these models occurs independently of SG formation.

## Discussion

Stress granule formation and dynamics accelerate multiple neurodegenerative processes, and several proteins and transcripts contained within SGs are implicated in neurodegenerative disease (11,17,32,33). However, SGs also play neuroprotective roles in response to acute stressors (65,74–77). Here we took advantage of a viral peptide, nsP3, to specifically inhibit SG formation in a variety of model systems without impacting ISR pathways or global protein translation, which has been a potential confounder in prior studies (17,34–37). Although nsP3-WT consistently inhibited SG formation, it was unable to alleviate neurodegenerative pathology in ALS/FTD and FXTAS models. Instead, inhibiting SG formation largely had no effect or enhanced toxicity, suggesting that SGs may play important protective roles in neurodegenerative cascades.

Many neurodegeneration-associated proteins (e.g. TDP43 and Tau) can associate with SG markers in patient tissues (66,78–81). Given their LLPS properties and the presence of SG markers in pathological inclusions, SG formation is thought to promote inclusion formation in multiple neurodegenerative diseases (82,83). Indeed, promoting SG formation in the absence of stress through use of optogenetic tools that drive G3BP dimerization is sufficient to trigger formation of inclusions (33). However, we observed TDP43 to readily aggregate during acute stress even when SG formation was inhibited (Supplementary Figure S5B). This is consistent with other studies showing TDP43 and Tau form aggregates independently of SG formation (65,77,82). How then might SGs mediate neurodegenerative cascades?

Formation of TDP43 and polyglutamine aggregates can be protective rather than detrimental to survival of cultured neurons (12,53), and sequestration of mutant FUS to stress granules reduces levels of toxic species in the cytoplasm (75). Thus, SGs may serve to buffer accumulation of toxic oligomeric species which drive neurodegeneration. In this scenario, selective inhibition of SG formation would be expected to enhance accumulation of these proteins in their most toxic forms, which would enhance neuronal death as we observe here. Consistent with this model, the low complexity domains that allow SG proteins to interact have recently been shown to promote non-fibrillar elastic solids over time, while fibrillar-like structures typically form outside of these granules (PREPRINT Alshareedah *et al.bioRxiv*, 2023).

Both the unfolded protein response and heat shock response are activated in multiple neurodegenerative contexts—typically in association with elevated levels of p-eIF2α and reductions in global protein synthesis (18,84–87). Yet, SGs are not typically observed within neurodegenerative tissue samples at autopsy (18). While there may be technical reasons for this (88), perhaps the lack of SGs in pathologic samples reflects a failure in neurons to respond to chronic stress. In this scenario, pathologic aggregates of proteins like TDP43 or DPRs are remnants of earlier protective SG formation induced by cellular stress. In this study, preventing SG formation in response to initial stressors may accelerate induced cell death at earlier stages in the pathogenic process.

A beneficial role for SGs in neurodegenerative disease is supported by the fact that age is the most common risk factor for disease. Aging is associated with enhanced gene expression, dysregulated proteostasis, and increased oxidative damage, all of which exist as chronic activators of the integrated stress response (reviewed in (89). Further, many proteins that are involved in neurodegeneration are normally required for proper SG dynamics. Loss of TDP43 inhibits robust SG assembly, while ALS/FTD causing *TIA1* mutations prevent SG disassembly (54,79). Lastly, multiple studies have shown that overexpressing mutant, aggregate-prone peptides in a variety of model systems can elicit the ISR within short timeframes (23,84,90–94). Despite this accumulation of stressors and noted increases in p-eIF2α in neurons with age, SG formation efficiency appears to depreciate in older neurons and senescent cells (54,95,96). In this context, our findings suggest that inhibiting global translation in and of itself may be insufficient to combat aging-inducing insults, and SGs may play essential roles in promoting neuronal viability.

We and others have noted that cells pre-conditioned with acute or chronic stress exhibit impaired stress responses to exogenous stressors (e.g. sodium arsenite), due to elevated levels of the eIF2α phosphatase, GADD34 (97–99). If neurons within neurodegenerative tissue are chronically under stress, they may lose their ability to continually form and reform SGs in response to new stress events. Thus, neurodegeneration may not be the result of an overactive stress response, but rather, the inability to elicit a robust or consistent stress response over time (100). This is supported by studies showing loss of GADD34 enhances survival and delays neurodegeneration in various ALS model systems, and loss of PERK accelerates disease progression, while pharmacologically upregulating heat shock and unfolded protein response pathways significantly improves outcomes in ALS models (34,35,86,101–103). A more recent study has also shown that ISR inhibition via eIF2B activators enhances disease progression in ALS models (104). In the context of our own work, these studies support a predominantly protective role for the integrated stress response in neurodegeneration.

This study has some limitations. First, it relies on overexpression disease models across relatively short time frames. Thus, these studies do not recapitulate the gradual accumulation of insults over months to years as likely occurs in neurodegeneration in humans. Second, our approach to prevent SG formation in these neurodegenerative models, while novel, has its own caveats: SG inhibition is incomplete in our cellular model systems, and the nsP3-31 peptide itself appears in some contexts to have mild intrinsic toxicity, regardless of whether it can bind G3BP/rin or not (Figure 3). Third, we were unable to control for expression levels across our three experimental groups in *Drosophila*, with nsP3-WT consistently expressed at lower levels compared to the mApple and nsP3-mut controls. This may explain the modest rescue we observed in our serial heat shock experiments in G4C2 expressing flies (Figure 6B, right). Thus, future studies using *in vivo* mammalian systems and orthogonal approaches are needed to ascertain how generalizable these findings are to other contexts and settings.

Despite these caveats, our observations have potentially important implications for therapy development in neurodegenerative disease. If the ISR is an active participant in most neurodegenerative settings, then defining the distinction between SG formation and ISR induced translational repression and transcriptional activation within different neurodegenerative contexts will be important next steps in allowing us to target the correct nodes in these pathologic cascades. We propose that elucidating these nodes and defining their broader impact across the full time-course of neurodegenerative disease should both reveal novel therapeutic targets and help us avoid disruption of intrinsic neuroprotective cascades that may currently mitigate the severity and progression in these challenging conditions.

### Ethics statement

All vertebrate animal work was approved by the Committee on the Use and Care of Animals (UCUCA) at the University of Michigan, and the Louisiana State University Health Sciences Center at Shreveport's Animal Care and Use Committee (ACUC). All experiments were performed in accordance with UCUCA and ACUC guidelines. Rats (*Rattus norvegicus*) used for primary neuron collection were housed singly in chambers equipped with environmental enrichment. Rats used for *in vivo* studies were housed with the dam until weaning at three weeks of age. Thereafter, they were housed in pairs by gender. All studies were designed to minimize animal use. Rats were cared for by the Unit for Laboratory Animal Medicine at the University of Michigan or veterinary specialists at Louisiana State University; all individuals were trained and approved in the care and long-term maintenance of rodent colonies, in accordance with the NIH-supported Guide for the Care and Use of Laboratory Animals. All personnel handling the rats and administering euthanasia were properly trained in accordance with the UM Policy for Education and Training of Animal Care and Use Personnel. Euthanasia was fully consistent with the recommendations of the Guidelines on Euthanasia of the American Veterinary Medical Association.

## Supplementary Material

gkae655_Supplemental_File

## Data Availability

The data underlying this article are available in the article and in its online supplementary material.
