# Supplementary material for: Stress granule formation helps to mitigate neurodegeneration
Source: Nucleic Acids Res. 2024 Aug 6;52(16):9745–59. doi: 10.1093/nar/gkae655 (PMC11381325; doi:10.1093/nar/gkae655)
Supplement: gkae655_Supplemental_File [file gkae655_supplemental_file.pdf]

Supplementary Figure 1

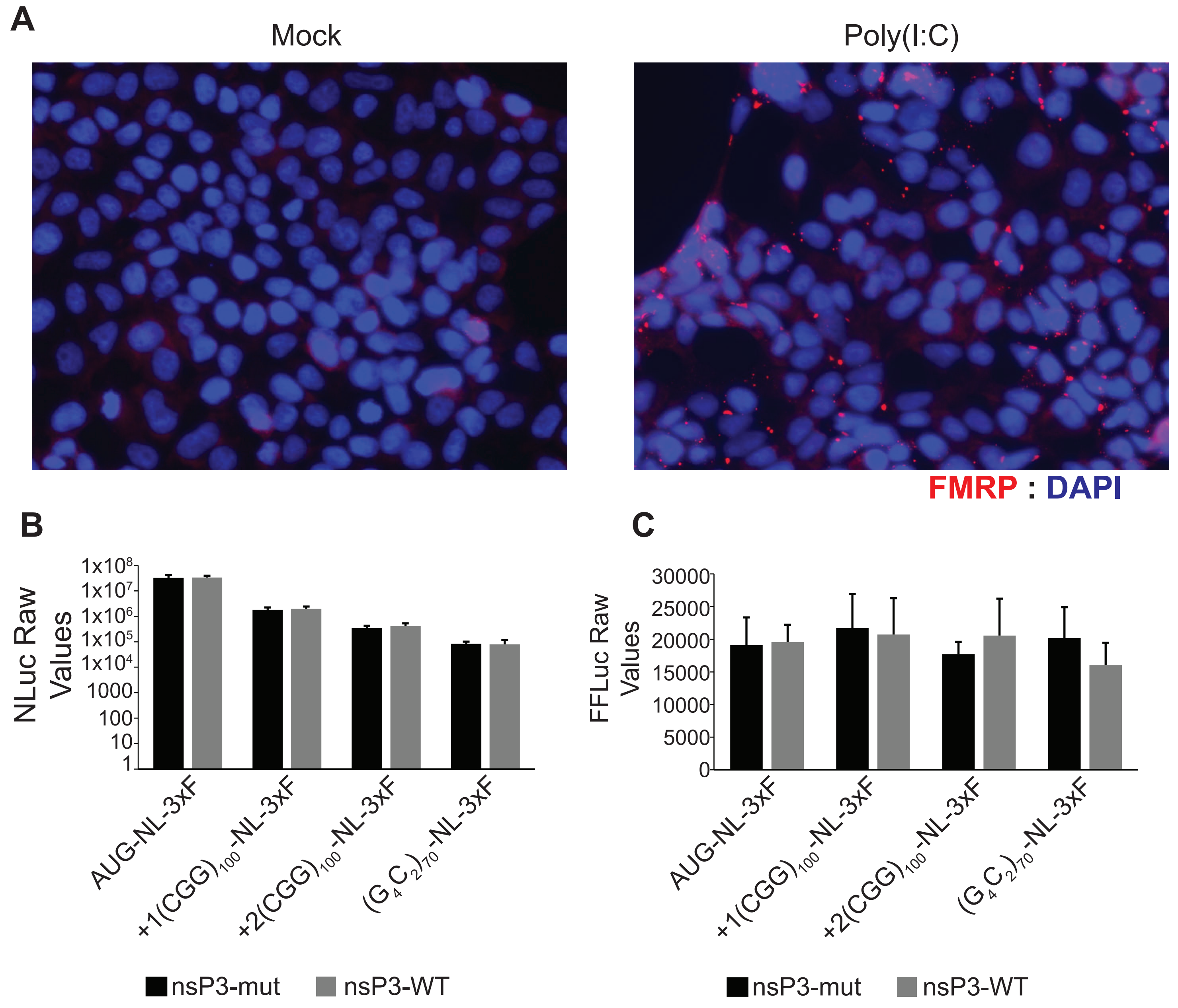

Supplementary Figure 1. nsP3-WT inhibits SG formation but has no impact on translation. A) Representative images of HEK 293Ts either mock or poly(I:C) transfected. FMRP= SG marker. B) Raw Nluc and C) FFluc values for experiment show in Figure 1F.

Supplementary Figure 2

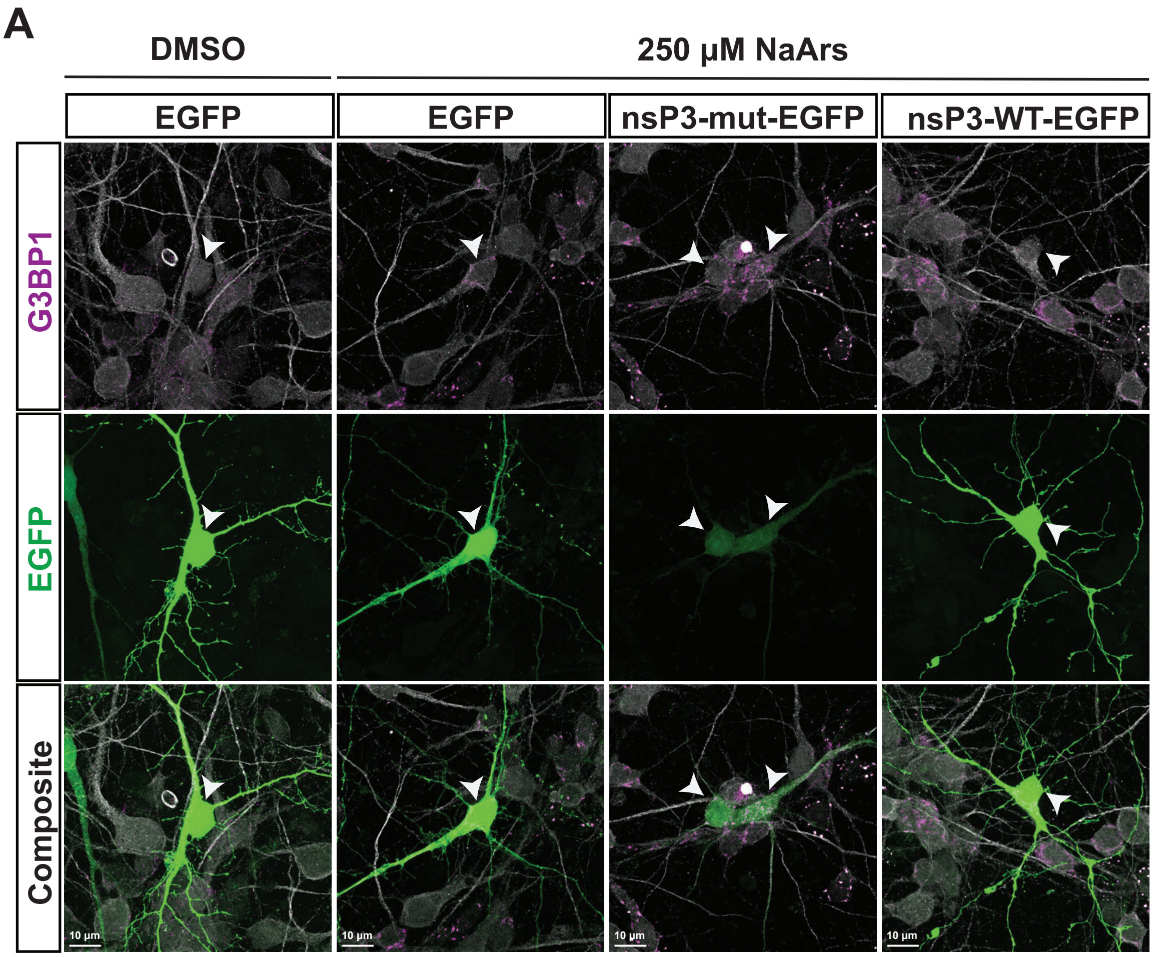

Supplementary Figure 2. nsp3 reduces stress granule formation in neurons. (A) Primary rat cortical neurons were transfected with EGFP, nsP3-WT-EGFP, or nsP3-mut-EGFP. 24h later, cells treated with vehicle (DMSO) or 250μM NaArs for 30 min prior to immunostaining for the stress granule marker G3BP1. Arrows point to transfected cells.

Supplementary Figure

3

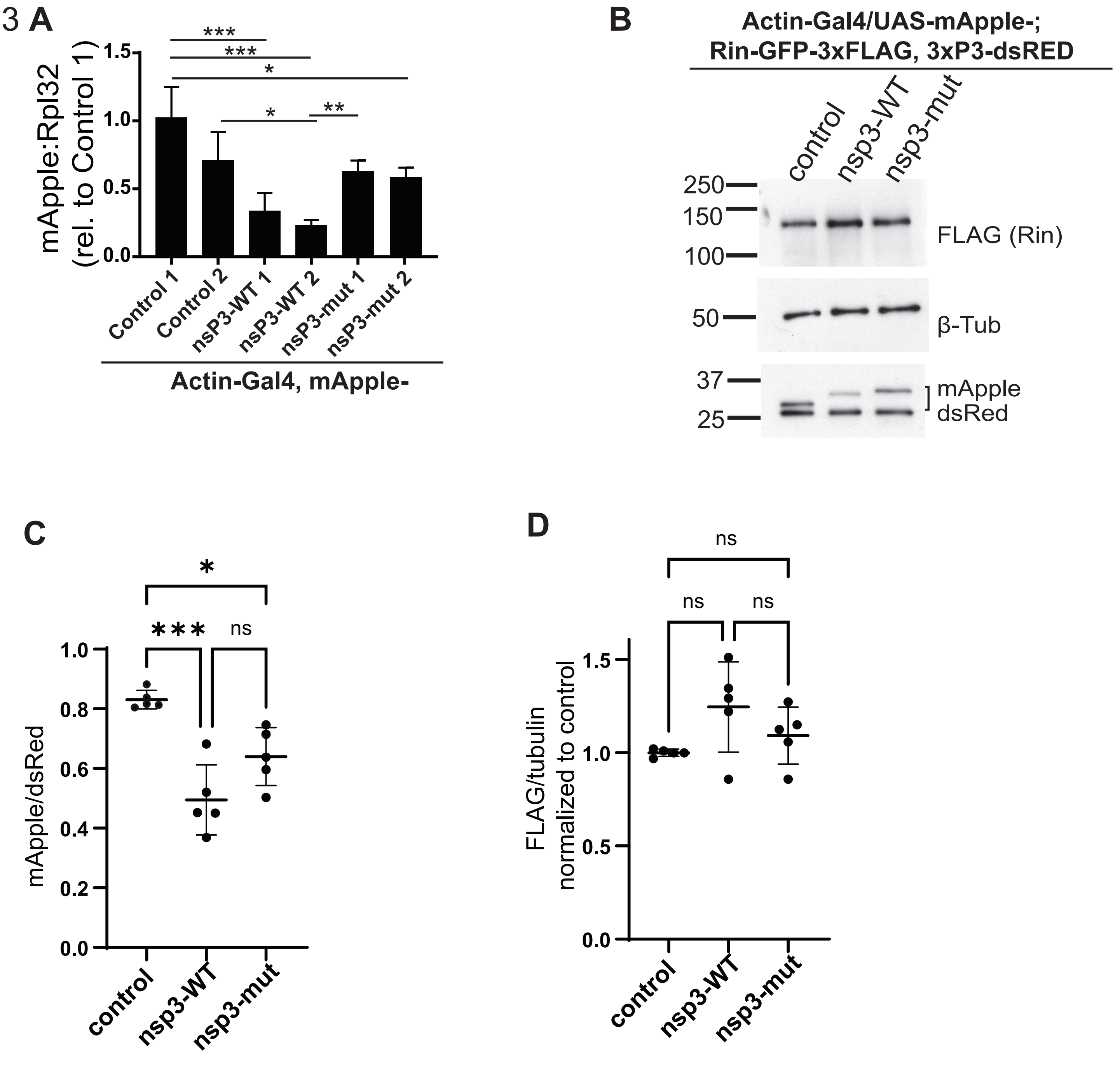

Supplementary Figure 3: Characterization of nsP3 in Drosophila. A) mRNA levels in Actin-Gal4, mApple-control, nsP3-WT, or nsP3-mut expressing 3rd instar larvae relative to Rpl32. Bars represent mean +/- standard deviation. One-way ANOVA with Tukey's multiple comparisons test, n=3 $*p<0.0125$, $**p<0.01$. B) Representative western blot of lysates from Actin-Gal4, Rin-GFP (115 kDa) + mApple-control (27 kDa), nsP3-WT (30.7 kDa), or nsP3-mut (30.9 kDa) *Drosophila* heads (note, Rin-GFP flies also contain 3xPs-dsRED (26.8 kDa) recognized by the antibody that binds mApple. B-tubulin= loading control. (C-D) Quantification of mApple and D) Flag signal in (B). Bars represent mean+/- standard deviation, n=5. Flag recognizes rin-sfGFP-3xFlag.

Supplementary Figure

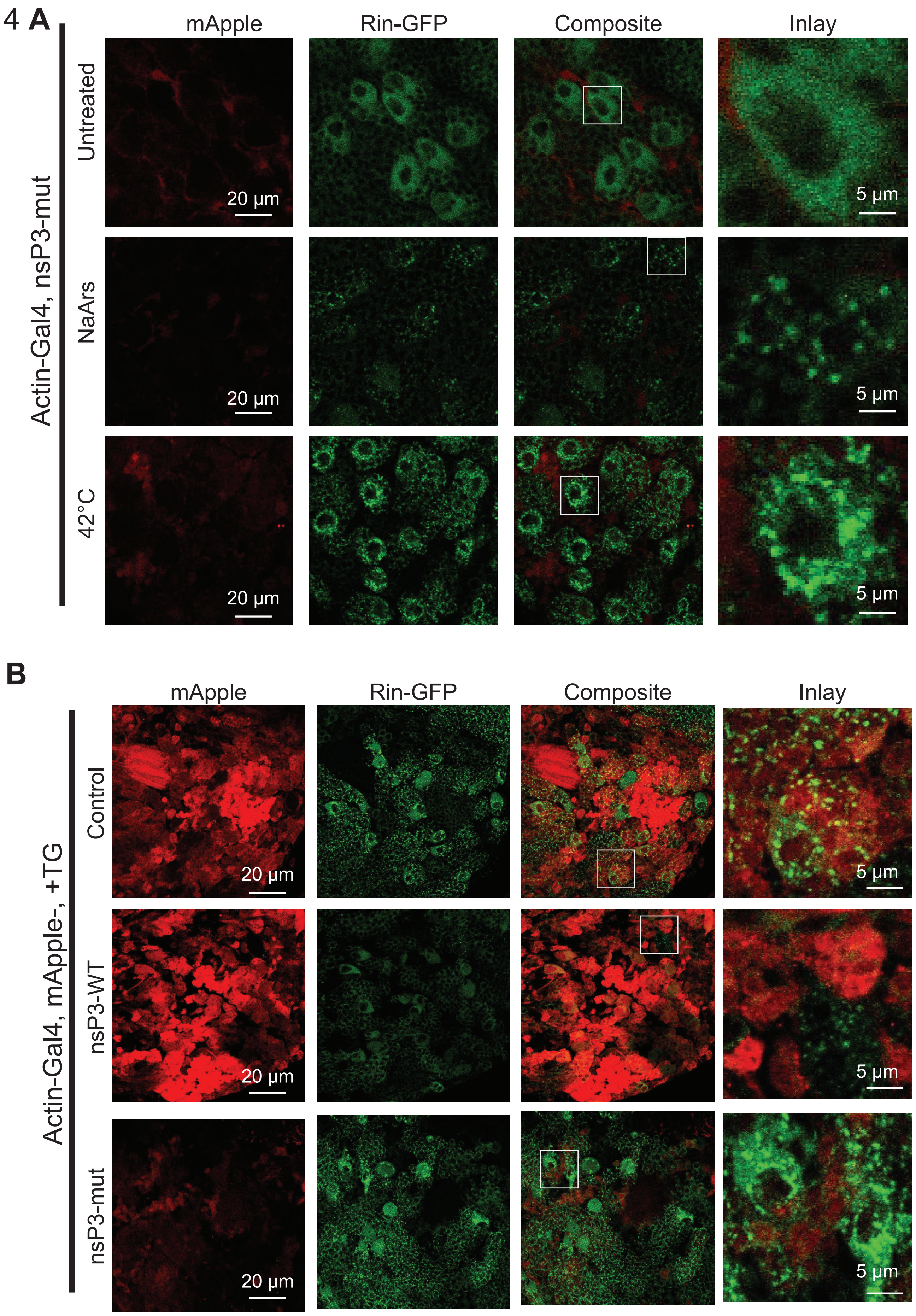

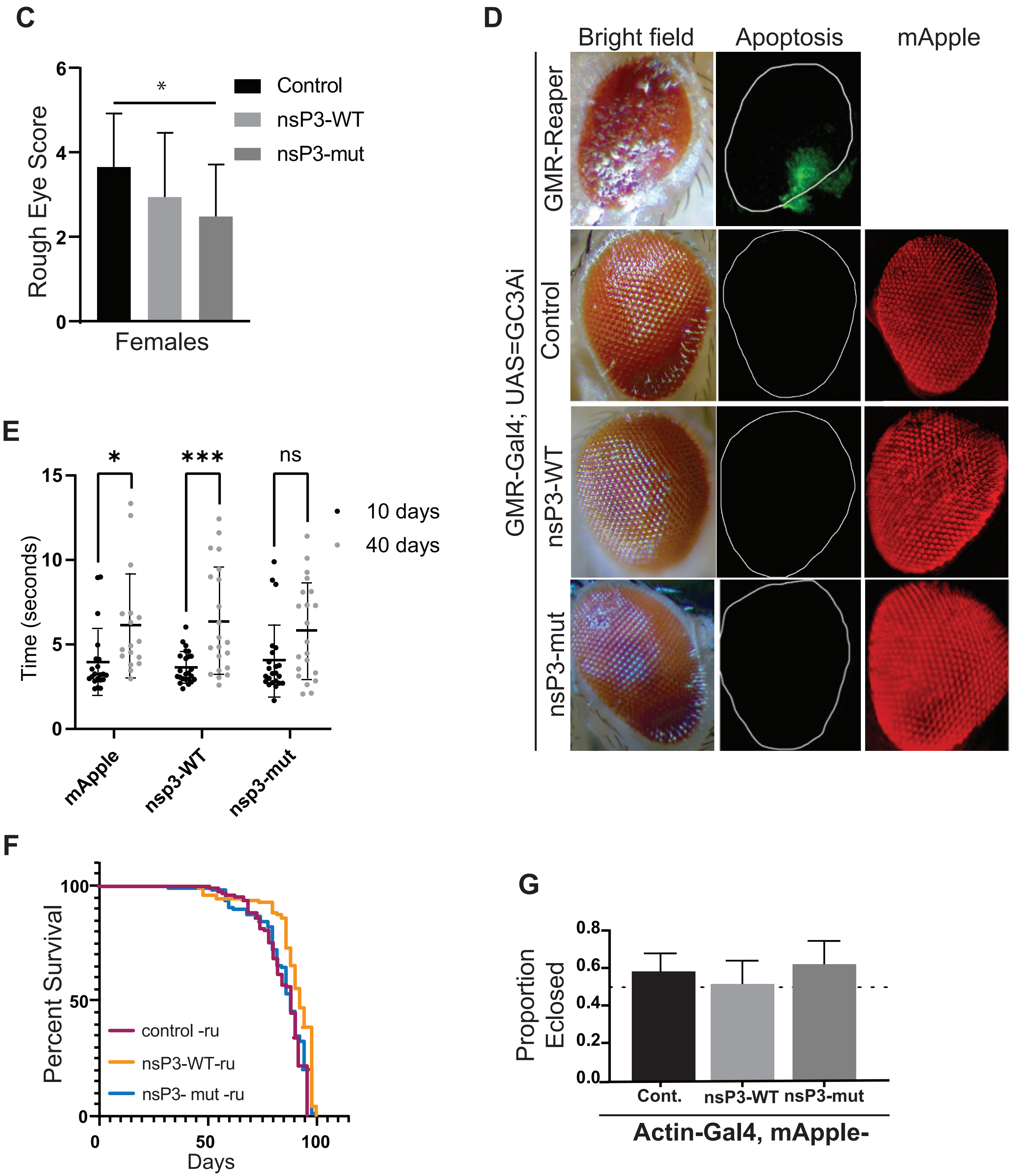
C
Rough Eye Score
6
4
2
0
*
Control
nsP3-WT
nsP3-mut
Females
D
Bright field
Apoptosis
mApple
GMR-Gal4; UAS=GC3Ai
GMR-Reaper
Control
nsP3-WT
nsP3-mut
E
Time (seconds)
15
10
5
0
*
***
ns
10 days
40 days
mApple
nsp3-WT
nsp3-mut
F
Percent Survival
100
50
0
control -ru
nsP3-WT-ru
nsP3- mut -ru
0
50
100
Days
G
Proportion Eclosed
0.8
0.6
0.4
0.2
0.0
Cont.
nsP3-WT
nsP3-mut
Actin-Gal4, mApple-

Supplementary Figure 4: nsP3 prevents SG formation in larval Drosophila brains. A) Representative images of 3rd instar larval brains expressing rin-sfGFP, and Actin-Gal4, nsP3-mut either untreated, or 1hr at 42C or 2hrs in 500uM NaArs. B) Representative images of 3rd instar larval brains expressing rin-sfGFP, Actin-Gal4 and either mApple control, nsP3-WT or nsP3-mut in the presence of 10uM thapsigargin (TG) for 90 minutes. C) Quantification of rough eye phenotypes of female flies expressing GMR-Gal4; EGFP-TDP43 and either control, nsP3-WT, or nsP3-mut (Control n= 24, nsP3-WT n= 22, nsP3-mut n= 22). One-way ANOVA and Tukey's multiple comparisons test. *p<0.05. D) Representative images of fly eyes coexpressing GMR-Gal4; UAS-GC3Ai with mApple, nsP3-WT, nsP3-mut, or GMR-reaper (positive control for apoptosis). GC3Ai is a modified GFP that only fluoresces when cleaved by caspases during apoptosis (Schott, Ambrosini et al. 2017). E) Climbing assay depicted in Figure 4E showing differences in climbing within genotypes with age. F) Survival assay of Tub5-GS, mApple control, nsP3-WT, or nsP3-mut flies without RU-486 at 24C. (control n= 91, nsP3-WT n= 89, nsP3-mut n= 92). Log-rank Mantel-Cox test. G) Bar graph represents proportion of eclosed flies from Actin-Gal4/Cyo crossed to homozygous mApple control, nsP3-WT, or nsP3-mut flies that were mApple+, +/- 95% C.I.s. Chi Square Analysis, (control n=101, nsP3-WT n= 66, nsP3-mut n=58). Dotted line is expected assuming no synthetic lethality caused by gene expression.

Supplementary Figure 5

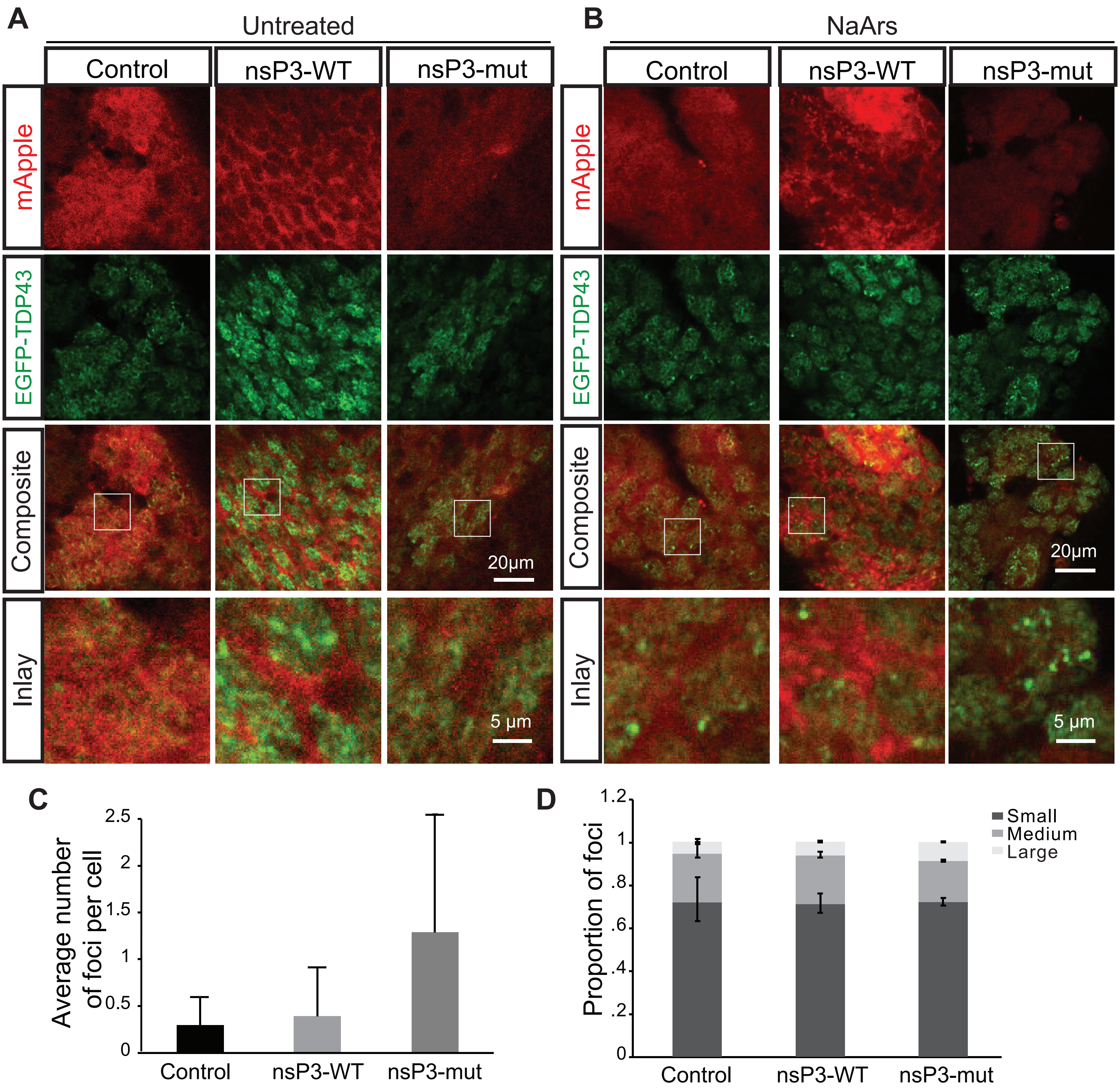

Supplementary Figure 5. nsP3-WT has no impact on TDP43 foci formation. A-B) Representative images of 3rd instar larval brains expressing Asense-Gal4; EGFP-TDP43, and mApple control, nsP3-WT, or nsP3-mut, incubated for 2 hrs untreated (A) or in 500uM NaArs (B). C) Quantification of average number of foci/cell/image (n=images: control n= 10, nsP3-WT n=14, nsP3-mut n=12) in (B). Bars represent mean +/- standard deviation. Multiple T-tests, no significance. D) Quantification of foci size distribution from images in (B). Bars represent proportion of total foci +/- 95% confidence intervals. Chi-square analysis, (control n= 71, nsP3-WT n=221, nsP3-mut n=479), not significant.

Supplementary Figure 6

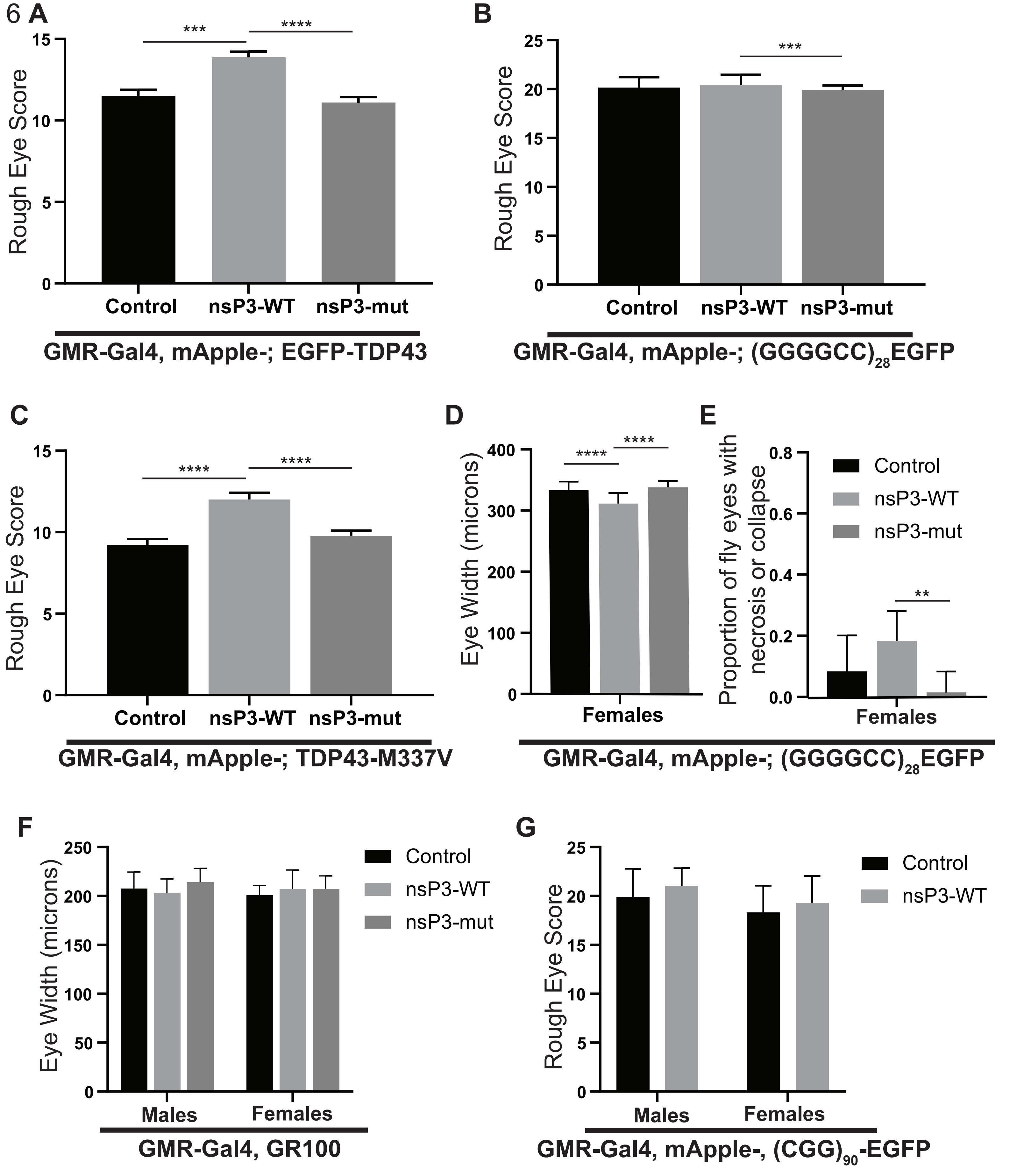

Supplementary Figure 6: nsP3 enhances toxicity in ALS fly models. A-C) Quantification of female GMR-Gal4/ mApple control, nsP3-WT, or nsP3-mut + A) TDP43TDP43-EGFP (control n= 60, nsP3-WT n= 52, nsP3-mut n= 88) ****p<0.0001, B) (GGGGCC)28-EGFP (control n=48, nsP3-WT n=82, nsP3-Mut=71) **p<0.01, and C) TDP43-M337V (control n= 37, nsP3-WT n= 38, nsP3-mut n= 54) ****p<0.0001 rough eye phenotype. A-C) One-way ANOVA with Tukey's multiple comparison tests. Quantification of (GGGGCC)28-EGFP female D) eye width (control n= 23, nsP3-WT n= 22, nsP3-mut n= 25) ****p<0.0001, and E) proportion of necrosis positive female eyes (control n=48, nsP3-WT n=82, nsP3-Mut=71). F) Quantification of GR100 eye width (control male n= 24, female n= 12; nsp3-WT male n= 17, female n= 10; nsP3-mut male n= 11, female n= 11). D & F: Two-way ANOVA (see Figure 6F), and Tukey's multiple comparison tests. E: Fischer's exact test with Bonferonni correction for multiple comparisons. ***p<0.001. G) Quantification of GMR-Gal4, (CGG)90-EGFP, mApple-x rough eye phenotype (males control n= 45, nsP3-WT n= 14; females control n= 41, nsP3-WT= 23). Two-way ANOVA with Sidak's multiple comparisons test.

Supplementary Figure 7

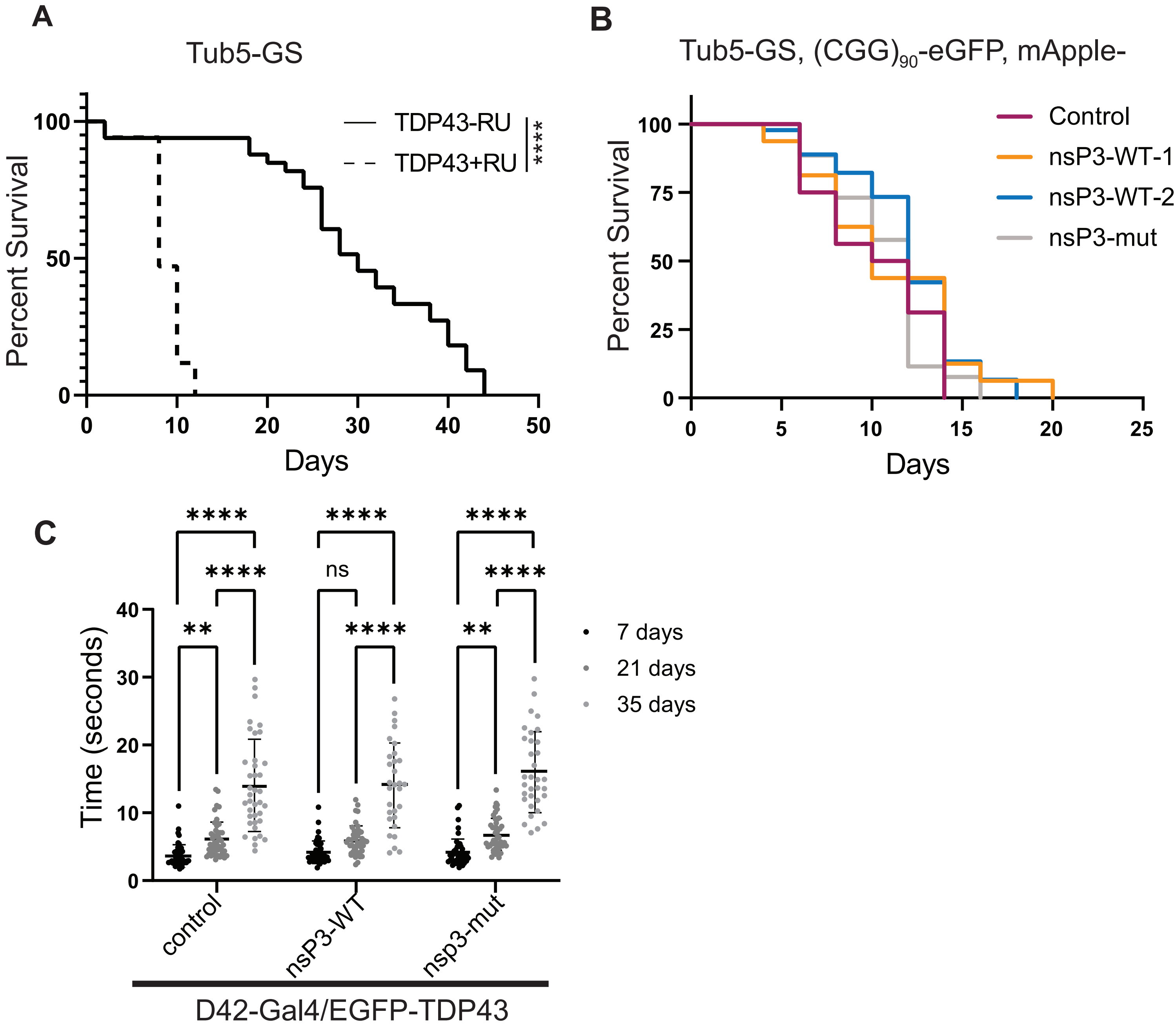

Supplementary Figure 7. nsP3 has no effect on neurodegenerative phenotypes in fly models. A) Survival assay of Tub5-GS, TDP43-GFP +/- RU B) Survival assay of Tub5-GS, (CGG)90-EGFP, crossed to mApple control, nsP3-WT, or nsP3-mut. (control n= 16, nsP3-WT-1 n=16, nsP3-WT-2 n=45, nsP3-mut n=26). Log-rank Mantel-Cox test with Bonferroni corrections for multiple comparisons. C) Climbing assay depicted in Figure 6C showing differences in climbing within genotypes with age.

Supplementary Table 1: Plasmid and Primer Sequences used in this study.

**Insert name**	**Sequence**
mApple	GCGGCCGccaccATGGTGAGCAAGGGCGAGGAGAATAACATGGCCATCATCAAGGAGTTCATGCGCTTC<br>AAGGTGCACATGGAGGGCTCCGTGAACGGCCACGAGTTCGAGATCGAGGGCGAGGGCGAGGGCCGCC<br>CCTACGAGGCCTTTCAGACCGCTAAGCTGAAGGTGACCAAGGGTGGCCCCCTGCCCTTCGCCTGGGAC<br>ATCCTGTCCCCTCAGTTCATGTACGGCTCCAAGGTCTACATTAAGCACCCAGCCGACATCCCCGACTAC<br>TTCAAGCTGTCCTTCCCCGAGGGCTTCAGGTGGGAGCGCGTGATGAACTTCGAGGACGGCGGCATTAT<br>TCACGTTAACCAGGACTCCTCCCTGCAGGACGGCGTGTTCATCTACAAGGTGAAGCTGCGCGGCACCA<br>ACTTCCCCTCCGACGGCCCCGTAATGCAGAAGAAGACCATGGGCTGGGAGGCCTCCGAGGAGCGGAT<br>GTACCCCGAGGACGGCGCCCTGAAGAGCGAGATCAAGAAGAGGCTGAAGCTGAAGGACGGCGGCCAC<br>TACGCCGCCGAGGTCAAGACCACCTACAAGGCCAAGAAGCCCGTGCAGCTGCCCGGCGCCTACATCGT<br>CGACATCAAGTTGGACATCGTGTCCCACAACGAGGACTACACCATCGTGGAACAGTACGAACGCGCCG<br>AGGGCCGCCACTCCACCGGCGGCATGGACGAGCTGTACAAGTAAGAATTCTGCAGTCGACGGTACCTC<br>TAGA
mApple-WT nsP3	GCGGCCGccaccATGGTGAGCAAGGGCGAGGAGAATAACATGGCCATCATCAAGGAGTTCATGCGCTTC<br>AAGGTGCACATGGAGGGCTCCGTGAACGGCCACGAGTTCGAGATCGAGGGCGAGGGCGAGGGCCGCC<br>CCTACGAGGCCTTTCAGACCGCTAAGCTGAAGGTGACCAAGGGTGGCCCCCTGCCCTTCGCCTGGGAC<br>ATCCTGTCCCCTCAGTTCATGTACGGCTCCAAGGTCTACATTAAGCACCCAGCCGACATCCCCGACTAC<br>TTCAAGCTGTCCTTCCCCGAGGGCTTCAGGTGGGAGCGCGTGATGAACTTCGAGGACGGCGGCATTAT<br>TCACGTTAACCAGGACTCCTCCCTGCAGGACGGCGTGTTCATCTACAAGGTGAAGCTGCGCGGCACCA<br>ACTTCCCCTCCGACGGCCCCGTAATGCAGAAGAAGACCATGGGCTGGGAGGCCTCCGAGGAGCGGAT<br>GTACCCCGAGGACGGCGCCCTGAAGAGCGAGATCAAGAAGAGGCTGAAGCTGAAGGACGGCGGCCAC<br>TACGCCGCCGAGGTCAAGACCACCTACAAGGCCAAGAAGCCCGTGCAGCTGCCCGGCGCCTACATCGT<br>CGACATCAAGTTGGACATCGTGTCCCACAACGAGGACTACACCATCGTGGAACAGTACGAACGCGCCG<br>AGGGCCGCCACTCCACCGGCGGCATGGACGAGCTGTACAagtccggactcaGATCTCTGCCCCTGACCTTC<br>GGCGACTTCGACGAGCACGAAGTGGACGCCCTGGCCAGCGGCATCACATTCGGCGATTTTGACGACGT<br>GCTGAGGCTCTGAGaattctgcagtcgacGGTACCTCTAGA
mApple-F3A nsP3	GCGGCCGccaccATGGTGAGCAAGGGCGAGGAGAATAACATGGCCATCATCAAGGAGTTCATGCGCTTC<br>AAGGTGCACATGGAGGGCTCCGTGAACGGCCACGAGTTCGAGATCGAGGGCGAGGGCGAGGGCCGCC<br>CCTACGAGGCCTTTCAGACCGCTAAGCTGAAGGTGACCAAGGGTGGCCCCCTGCCCTTCGCCTGGGAC<br>ATCCTGTCCCCTCAGTTCATGTACGGCTCCAAGGTCTACATTAAGCACCCAGCCGACATCCCCGACTAC<br>TTCAAGCTGTCCTTCCCCGAGGGCTTCAGGTGGGAGCGCGTGATGAACTTCGAGGACGGCGGCATTAT<br>TCACGTTAACCAGGACTCCTCCCTGCAGGACGGCGTGTTCATCTACAAGGTGAAGCTGCGCGGCACCA<br>ACTTCCCCTCCGACGGCCCCGTAATGCAGAAGAAGACCATGGGCTGGGAGGCCTCCGAGGAGCGGAT<br>GTACCCCGAGGACGGCGCCCTGAAGAGCGAGATCAAGAAGAGGCTGAAGCTGAAGGACGGCGGCCAC<br>TACGCCGCCGAGGTCAAGACCACCTACAAGGCCAAGAAGCCCGTGCAGCTGCCCGGCGCCTACATCGT<br>CGACATCAAGTTGGACATCGTGTCCCACAACGAGGACTACACCATCGTGGAACAGTACGAACGCGCCG<br>AGGGCCGCCACTCCACCGGCGGCATGGACGAGCTGTACAagtccggactcaGATCTCTGCCCCTGACCgctG<br>GCGACTTCGACGAGCACGAAGTGGACGCCCTGGCCAGCGGCATCACAgctGGCGATTTTGACGACGTGC<br>TGAGGCTCTGAGaattctgcagtcgacGGTACCTCTAGA
mApple 5’	GGCCACGAGTTCGAGATCG
mApple 3’	GGACAGCTTGAAGTAGTCGGG
RPL32 5’	CGGATCGATATGCTAAGCTGTCG
RpL32 3’	GCATGAGCAGGACCTCCAG

Supplementary Table 2: Transgenic fly lines used in this study.

Genotype	Figures	Source
;UAS-mApple/CyO	Fig. 4C-G, Fig. 5, Fig. 6 Supp. Fig. 3, Supp. Fig. 4 C-G, Supp. Fig 5, Supp. Fig 6., Supp. Fig. 7	This study
;UAS-mApple-nsP3-WT 2//CyO	Fig. 4C-G, Fig. 5, Fig. 6 Supp. Fig. 3, Supp. Fig. 4 C-G, Supp. Fig 5, Supp. Fig 6., Supp. Fig. 7	This study
;UAS-mApple-nsP3-mut//CyO	Fig. 4C-G, Fig. 5, Fig. 6 Supp. Fig. 3, Supp. Fig. 4 C-G, Supp. Fig 5, Supp. Fig 6., Supp. Fig. 7	This study
;UASz-mApple-nsP3-WT/CyO	Fig. 4B, Supp. Fig. 4A-B	This study
;UASz-mApple-nsP3-mut//CyO	Fig. 4B, Supp. Fig. 4A-B	This study
UAS-EGFP-TDP43	Fig 5A-B, Fig 6A, C, Supp. Fig. 5, Supp. Fig 6A, Supp. Fig. 7A and C	This study
GMR-Gal4, UAS-CGG90-EGFP-BD/CyO	Supp. Fig. 6G, Supp Fig. 7B	(Jin et al. 2003)
w[1118];P{{y[+t7.7]w[+mC]=UAS-poly-GR.PO-100}attP40	Supp. Fig. 6F	(Mizielinska and Isaacs 2014)
; UAS-(G4C2)28-EGFP	Fig. 5D-F, Supp. Fig. 6B, D-E	(He et al. 2020)
;;UASt-TDP43-M337/Tm3	Fig. 5C, Supp. Fig. 6C	(Ritson et al. 2010)
FlyFos0289429pRedFlp-Hgr)(rin[36711]::2XTY1-SGFP-V5-pre-TEV-BLRP-3XFLAG)dFRT	Fig. 4B, Supp. Fig. 4A-B	(Sarov et al. 2016)
Actin-Gal4	Fig. 4B, Supp. Fig. 3, Supp. Fig. 4A-B, G	
GMR-Gal4	Fig. 4C-D, Fig. 5, Supp. Fig. 4D, Supp. Fig. 6	BDSC 8605
Tub5-GeneSwitch	Fig. 4F-G, Fig. 6A-B, Supp. Fig. 4F	(Osterwalder et al. 2001)
D42-Gal4	Fig. 4E, Fig. 6C, Supp. Fig. 4E, Supp Fig 7C	BDSC8816
